# TiN nanotube supported Ni catalyst Ni@TiN-NTs: experimental evidence of structure–activity relations in catalytically hydrolyzing ammonia borane for hydrogen evolution†

**DOI:** 10.1039/d0ra06920e

**Published:** 2020-10-08

**Authors:** Yawei Liu, Jun Zhang, Quanxing Liu, Xiang Li

**Affiliations:** Chemical Engineering & Pharmaceutics School, Henan University of Science & Technology Luoyang 471023 China j-zhang@126.com

## Abstract

With commercial TiO_2_ as the precursor, titanium nitride nanotubes (TiN-NTs) were fabricated through a hydrothermal – ammonia nitriding route, and next non-noble metal nanosized Ni particles were evenly and firmly anchored on the surface of the TiN-NTs *via* a PVP-mediated non-aqueous phase reduction–deposition strategy, to obtain the supported catalyst Ni@TiN-NTs. The X-ray powder diffraction (PXRD), field emission scanning and transmission electron microscopy (FE-SEM/TEM) and specific surface area measurements were used to characterize and analyze the phase composition, surface microstructure and morphological features of the product. The catalytic activity of the Ni@TiN-NTs for hydrolyzing ammonia borane to generate hydrogen (H_2_) under different conditions was evaluated systematically. The results reveal that the as-fabricated TiN-NTs are composed of TiN and a small amount of TiN_*x*_O_*y*_ with the approximate molar atomic ratio of Ti to N at 1 : 1, existing as hollow microtubules with mean tube diameter of 130 nm and length of about 1 μm. *Via in situ* reduction and deposition, Ni nanoparticles can be uniformly anchored on the surface of TiN-NTs. The catalytic activities of Ni(*x*)@TiN-NTs with different Ni loading amounts are all higher than that of single metal Ni nanoparticles. The temperature has a positive effect on the catalytic activity of Ni(20)@TiN-NTs, and its total turnover frequency for hydrolyzing ammonia borane is 11.73 mol(H_2_) (mol Ni)^−1^ min^−1^, with an apparent activation energy of 52.05 kJ mol^−1^ at 303 K. After 5 cycles, the Ni(20)@TiN-NTs catalyst still maintains 87% of the initial catalytic activity. It could be suggested that these tactics can also be extended to the fabrication of other metal or alloy catalysts supported by TiN-NTs, with great application potential and development prospects.

## Abstract 2

Titanium nitride nanotubes (TiN-NTs) were fabricated using a hydrothermal – ammonia nitriding route, and non-noble metal nanosized Ni particles were anchored on the surface *via* a non-aqueous phase reduction–deposition strategy, to obtain the supported catalyst Ni@TiN-NTs.

## Graphical abstract

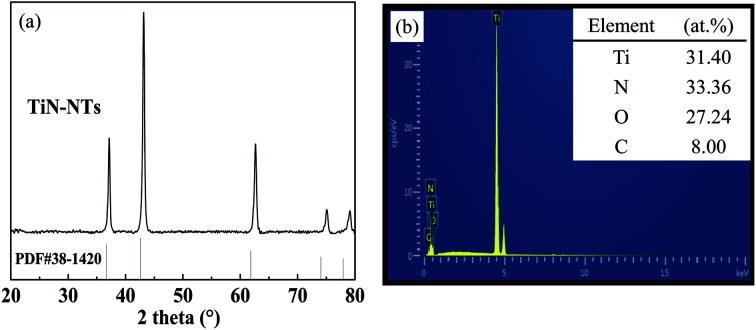

Research and development of hydrogen energy, recognized as a new green energy with high efficiency and eco-friendliness, is increasingly attracting extensive attention.^1,2^ Safe, convenient and controllable hydrogen storage/release tactics is the premise and basis for large-scale utilization of hydrogen energy, which is also the key factor restricting the rapid development of the hydrogen energy economy, still confronting much theoretical and technical challenges so far.^3–6^ Ammonia borane (AB, NH_3_BH_3_) is a typical representative of chemical hydrogen storage materials with a theoretical hydrogen storage capacity of 19.6 wt%, regarded as the compound with the highest hydrogen content found up to now.^7,8^ AB can exist in the solid state under the atmospheric environment, with the unique physicochemical properties such as nontoxicity, incombustiblity, no corrosion and no explosion so on. Moreover, AB can release hydrogen gas (H_2_) in a controllable manner *via* catalytic hydrolysis or alcoholysis, which is thus considered as an ideal hydrogen storage medium for fuel cells.^9^ It is well documented in current studies that most of the catalysts for hydrolyzing ammonia borane to release H_2_ are made up of precious metals acted as the main active ingredient.^10–13^ Although these catalysts hold good catalytic activity, the high price limits their large-scale application. Therefore, to seek new catalysts with high activity and low cost is of urgent practical demand, with the considerably potential R&D prospect. Titanium nitride (TiN), as a kind of multifunctional metal ceramic with some unique physicochemical properties, is causing wide concern in recent years.^14,15^ Due to inserting N atoms into the Ti metal lattice, the d-band holes increase and the Fermi energy level drops, rendering the similar surface properties of TiN with those of Pt-group noble metals.^16,17^ Thus, the TiN metal ceramic is endowed with high hardness, low density, high strength, corrosion resistance and good electrical conductivity, which has made it find lots of applications in the catalytic materials,^18^ electrode materials^19^ and other fields. In contrast, nanotube-like microstructured TiN, or TiN-NTs, possesses the relatively larger specific surface area and pore volume. Owing to the complicated multi-dimensional structure and charged network that are conducive to the directional and rapid electron conduction, the TiN-NTs gives particular edge as the catalyst carrier.^20–22^ Fabian and coworkers^23^ prepared the carbon-coated TiN nanotube array material, and used it as the cathode of the supercapacitor, finding that the cathode holds excellent reversibility for charge storage. Pan *et al.*^24^ loaded Pt onto TiN nanotubes and experimentally validated that its catalytic activity for oxygen reduction was significantly enhanced. The current research findings manifest that TiN with regular and ordered nanotube texture could engender obvious collaborative or synergistic effect with some metal atoms (*e.g.* Pt, Pd, *etc.*), thus generating more active sites on its surface, and effectively improving its catalytic ability. Based on the above cognition and analysis for the structure–activity relationship of the TiN-NTs, *via* elaborately designing the hydrothermal treatment-nitriding synthesis route, we first fabricated TiN-NTs microarchitectures with regular profiles; and then by way of the solvothermal reduction-deposition tactics that was *in situ* mediated by PVP, non-noble metal Ni nanoparticles were dispersed and immobilized on the TiN-NTs surface, to obtain highly efficient and low-cost Ni@TiN-NTs supported catalyst with no noble metals contained. Meanwhile, a set of feasible device and scheme were devised to scientifically evaluate the catalytic ability of Ni@TiN-NTs on hydrolyzing ammonia borane to release H_2_, and satisfactory results were obtained.

## 1 Experimental

### 1.1 Reagents and instruments

Titanium dioxide (TiO_2_, 99%), nitric acid (HNO_3_, 65%), sodium hydroxide (NaOH, 98%), ethylene glycol ((CH_2_OH)_2_, EG, 99%), nickel chloride (NiCl_2_·6H_2_O, 98%), hydrazine hydrate (N_2_H_4_·H_2_O, 85%), absolute ethyl alcohol (C_2_H_5_OH, 98%) and polyvinylpyrrolidone (PVP, *M*_w_ = 1 × 10^4^), are pure analytical reagents purchased from Shanghai Sinopharm Group and used directly without further purification. Ammonia borane (NH_3_BH_3_, referred to as AB) was prepared in our laboratory according to the method depicted in the related report.^25^ Based on our comprehensive analysis, the purity of as-prepared AB was determined to be no less than 98%.^9^ The water used in the whole experiment was doubly distilled water, whose conductivity (298 K) was less than 1.0 μS cm^−1^. The phase structure of the serial products was analyzed by X-ray powder diffraction (PXRD) (Advance-D8, Bruker, Germany). Their morphologies were minutely investigated by field emission scanning electron microscopy (FE-SEM) (JEM-2100, JEOL, Japan) and transmission electron microscopy (TEM) (JEM 2100, Hitachi, Japan). Specific surface area measurements were performed and recorded on the fully automated micropore analyzer (Autosorb-IQ-MP, Quantachrome, USA).

### 1.2 Fabriction of titanium nitride nanotubes (TiN-NTs)

Typically, 0.75 g TiO_2_ micropowder was added to 85 ml concentrated NaOH solution (10 mol l^−1^), followed by ultrasonic dispersion for 40 min to get a milky suspension, which was transferred into a 100 ml teflon-lined autoclave. After closely sealed, the autoclave was placed in a furnace and heated to 140 °C to hydrothermally react for 24 hours. Upon finishing the reaction, it was naturally cooled to room temperature. After decanting the supernatant from the autoclave, the remaining precipitate was transferred into 0.1 mol l^−1^ HNO_3_ solution. *Via* stirring and pickling for 2 hours, the precipitate was repeatedly washed with deionized water until neutral, prior to vacuum drying at 40 °C for 6 hours. Next, the precipitate was thermally treated in a program control furnace at 400 °C for 2 h, so as to be transformed to the anatase phase TiO_2_-NTs. Placing the TiO_2_-NTs in a tube furnace, followed by inletting nitrogen gas to the tube for 20 min to remove the air, the tube furnace was heated to 500 °C at a heating rate of about 5 °C min^−1^, staying at this temperature for 10 min. Afterwards, the inlet gas was switched to high purity ammonia with flow rate of 120 ml min^−1^, and the tube furnace was heated from 500 °C to 850 °C at the same heating rate. At the temperature (850 °C) the reaction in the furnace was maintained for 3 h, followed by the natural cooling to room temperature, and the resulting product, or titanium nitride nanotube particles (TiN-NTs), could be harvested.

### 1.3 Preparation of nanosized Ni particles and *in situ* loading on TiN-NTs

#### 1.3.1 Preparation of nanosized metal Ni powders

Under the nitrogen protection, into a 30 ml glass vial was injected with 8.60 ml EG, followed by addition of 0.0827 g NiCl_2_·6H_2_O and 0.0831 g PVP, *via* ultrasonic dispersion until the solids were fully dissolved. The vial was fixed in the water bath with the temperature raised to 60 °C, prior to injection of 0.40 ml N_2_H_4_·H_2_O under medium speed magnetic stirring. As the solution color turned purple, the pH of the solution was adjusted in the range of 10 to 11 by dropwise adding 1.0 mol l^−1^ NaOH, and the reaction was maintained for extra 30 min. After closing the reaction, by centrifuging and washing the precipitate with 95% ethanol for several times, and then drying it in vacuum at 40 °C for 2 h, the black metal Ni nanoparticles (Ni-NPs) were obtained.

#### 1.3.2 *In situ* loading of nanometer Ni on TiN-NTs

Under the nitrogen protection and ultrasonic treatment, into a 30 ml glass vial was injected with 8.60 ml EG, followed by addition of 0.0827 g NiCl_2_·6H_2_O and 0.0831 g PVP, until the complete dissolution. The newly prepared TiN-NTs powder (0.0816 g) was quickly added to the glass vial, with 30 min ultrasonic treatment to form a uniform suspension, followed by injection of 0.40 ml N_2_H_4_·H_2_O. The glass vial was set in the water bath with the temperature controlled at 60 °C, reacting for some time under ceaseless medium speed magnetic stirring. As the solution turned purple, by dropwise adding 1.0 mol l^−1^ NaOH the pH of the solution was adjusted to between 10 and 11, and the reaction continued for additional 30 min. On finishing the reaction, it was centrifugally separated, washed alternately with anhydrous ethanol and water for 5 times, and then the gray solid was collected and dried in vacuum at 40 °C for 4 h. Thus, the supported catalyst with a Ni load of 20% was obtained, which was denoted as Ni(20)@TiN-NTs.

According to the similar preparation procedure and conditions, by separately adjusting the dosage of Ni salt and reducing agent N_2_H_4_·H_2_O, the serial supported catalysts with Ni load of 16%, 12% and 8%, could be fabricated, which were accordingly labeled as Ni(16)@TiN-NTs, Ni(12)@TiN-NTs and Ni(8)@TiN-NTs, respectively.

### 1.4 Catalytic activity evaluation method

Under a certain temperature and pressure condition, hydrogen (H_2_) amount generated by catalytic hydrolysis of ammonia borane (AB) in unit time can be accurately measured, which could be used to evaluate the catalytic activity of the relevant catalysts. The catalytic hydrolysis test was conducted in a homemade pressure-resistant thick-wall glass reactor, which was placed in a constant temperature water bath with a temperature control accuracy of ±0.5 K. The upper outlet of the reactor is connected to a short-neck drying tube filled with soft, loose, breathable glass wool. The other end of the drying tube is linked to a vertical condenser, which is cooled by circulating ice water in order to capture volatile matters in real time. The cooled gas is fed into an absorption tube containing a certain amount of anhydrous calcium chloride to remove other gaseous impurities. The purified hydrogen is then introduced into the burette with fine scale to record the volume change of H_2_ gas in the tube per unit time.^26^ Once the hydrolysis reaction begins, the H_2_ volume generated at different time intervals needs to be accurately measured and recorded. In this work, the concentration of AB aqueous solution was set at 0.10 mol l^−1^, and the addition amount of catalyst (Ni-NPs, Ni(*x*)@TiN-NTs) powder was controlled at 5 mg per 10 ml AB solution.

## 2 Results and discussion

### 2.1 Phase constitution analysis

**Fig. 1:**
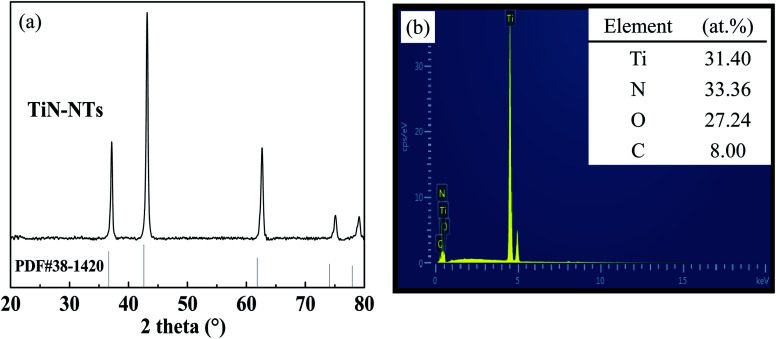
PXRD pattern (a) and EDS energy spectrum (b) of the TiN-NTs sample.

**Fig. 2:**
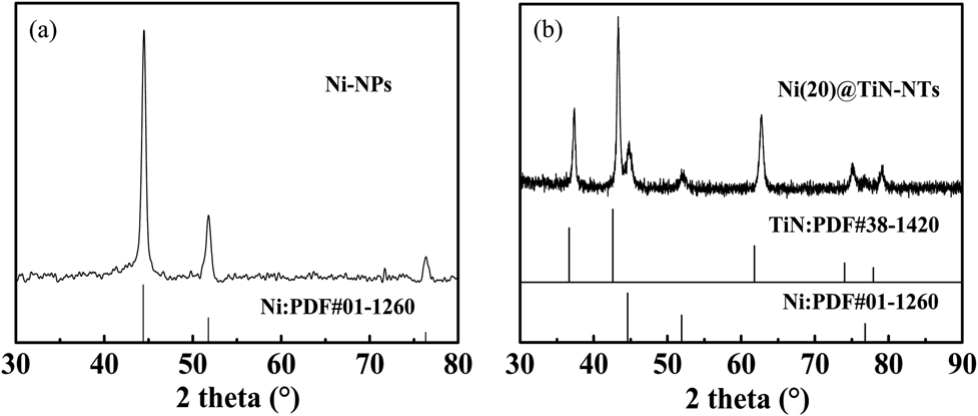
PXRD patterns of nanometer Ni-NPs (a) and Ni(20)@TiN-NTs (b).

The phase constitution of as-prepared TiN-NTs, Ni particles and the supported catalyst Ni(*x*)@TiN-NTs sample were analyzed by means of X-ray powder diffraction (PXRD), and the results are shown in Fig. 1 and Fig. 2, respectively. As can be seen from Fig. 1(a), for the TiN-NTs sample five distinct and sharp diffraction peaks appeared at 2*θ* = 36.8°, 42.6°, 62.2°, 74.4° and 78.5°. The comparison with the JCPDS standard card (PDF) reveals that these peaks could be basically assigned to the face centered cubic TiN (PDF 38-1420), indexed to the (111), (200), (220), (311) and (222) crystal planes of TiN, respectively. However, further detailed analysis finds that compared with the normative diffraction peak of TiN (PDF 38-1420), the five diffraction peaks of the as-fabricated TiN-NTs sample all indicate minor positive offset to different degrees in the direction of the high angle, and the mean deviation of 2*θ* angle is 0.13. According to the Bragg diffraction principle, the positive shift of diffraction peak may originate from the diminution of lattice parameters, which means that the TiN-NTs sample could contain other atoms smaller than the nitrogen atom radius.^27^ As is shown in the SEM-EDS spectrum of the TiN-NTs sample in Fig. 1(b), besides Ti and N elements, the TiN-NTs sample also contains a certain amount of O element. Through the analysis we can realize that the precursor of nitriding reaction process is TiO_2_-NTs, involving O atom, with the smaller atom radius (0.066 nm) than that of N atom (0.075 nm). During the nitriding reaction, O atoms in TiO_2_-NTs may not be completely replaced by N atoms, so some O atoms remain. Moreover, the atomic ratio of Ti and N elements is close to 1 : 1, which further proves that the TiN-NTs samples may be composed of abundant TiN, bits of amorphous titanium nitride oxide (TiN_*x*_O_*y*_) and low-value titanium oxide (Ti_*n*_O_2*n*−1_).^28^ There exist various bond forms such as Ti–N bond, Ti–O bond or O–Ti–N bond, which are conducive to increasing the catalytic performance of TiN-NTs.^29^

Fig. 2 represents the PXRD patterns of Ni(20)@TiN-NTs and the metal Ni micro–nano powder prepared in this work. As seen from Fig. 2(a), three peaks arise at 44.5°, 51.8° and 76.4°, which should be attributed to the three crystal planes (111), (200) and (220) of the face-centered cubic metal Ni (PDF 01-1260), respectively. No other unknown peaks are found, indicating that no oxides or hydroxides of nickel exist, and the purity of the as-prepared Ni powder is relatively high. From the PXRD pattern of Ni(20)@TiN-NTs (*cf.*Fig. 2(b)), it can be found that two sets of diffraction peaks can be discriminated, which could be attributed to those of TiN (PDF 38-1420) and metal Ni (PDF 01-1260), respectively. Based on the PXRD results it can be judged that the metal Ni nanoparticles have been supported on the surface of TiN-NTs.

### 2.2 Morphology analysis

**Fig. 3:**
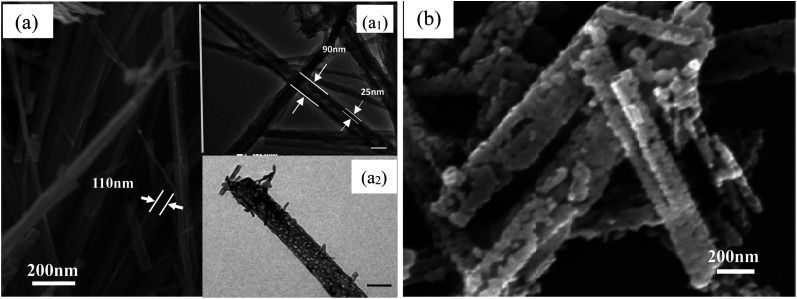
SEM image (a) and TEM image (a_1_ and a_2_) of the TiO_2_ samples; SEM image of the TiN-NTs samples (b). The scale bars in Fig.3 (a_1_ and a_2_) denote 100 nm.

The SEM and TEM images of the as-prepared TiO_2_ and TiN samples are displayed in Fig. 3. As clearly shown in Fig. 3(a), the intermediate product TiO_2_ presents microsized wire shape, with 120 nm mean diameter, more than 2 μm length and greater than 16 aspect ratio. The surface of the TiO_2_ micro-wires is relatively smooth, with visually compact texture, emerging as the mutually discrete state. However, after HNO_3_ pickling, the morphology of TiO_2_ significantly changed from micrometer line to nano-sized tube structure, just as shown in Fig. 3(a_1_). The diameter of TiO_2_ nanotubes after pickling is about 90 nm, which is slightly smaller than that before pickling. The thickness of the tube wall is about 25 nm, with a well-defined tube shape and obvious hollow structure. *Via* making a subtle observation, it can be seen that the surface of the nanotube has many finer micropores and tiny hairs (*cf.*Fig. 3(a_2_)). After nitriding treatment, TiO_2_ can be converted to TiN, and the corresponding shape also changes greatly (*cf.*Fig. 3(b)). The TiN product takes hollow short tubular shape with apparent mean diameter of 130 nm, and the length of the tube is obviously shorter than TiO_2_-NTs. The wall of the tube appears to be cross-linked by a large number of nano-particles and then curled into a tubular shape, with the tube surface full of pores. The unique structure of TiN-NTs facilitates the free conduction of internal electrons, while the larger specific surface area also provides more active sites and channels for the distribution and anchoring of active metal components.

**Fig. 4:**
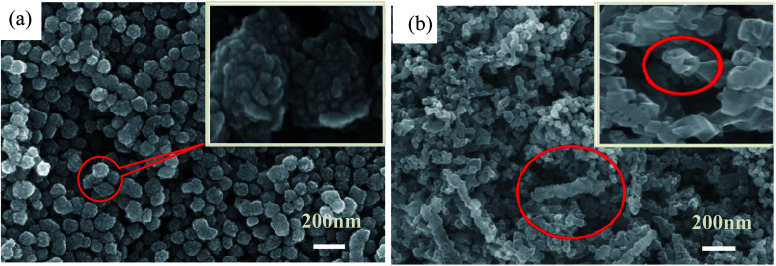
SEM image of Ni nanoparticles (a), and Ni(20)@TiN-NTs supported catalyst (b).

In contrast, the as-prepared metal Ni particles reveal relatively regular, disperse and uniform microsphere shape with mean size of 70 nm and less agglomeration, just as indicated in Fig. 4(a). But, it can be found from the inserted image that the surface of the Ni particles is quite rough, formed by aggregating of even smaller nanoparticles (10–20 nm). Fig. 4(b) demonstrates the surface profile of the as-fabricated Ni(20)@TiN-NTs. *Via* careful observation, it is easily identified that a bit of TiN nanotubes still exist, just become obviously shortened and irregular. The tiny Ni particles attached to the surface of TiN-NTs freely interlink with each other, resulting in the unordered, porous and diverse shape (see SEM-mapping Fig. S1 in ESI,† omitted here for saving space and simplicity). In consideration of the fabricating procedure, it could be speculated that ultrasonic and washing treatment should be responsible for the length shortening and channel collapse of the TiN-NTs microstructure.

### 2.3 Specific surface area measurement and analysis

#### 2.3.1 BET analysis of TiO_2_-NTs

**Fig. 5:**
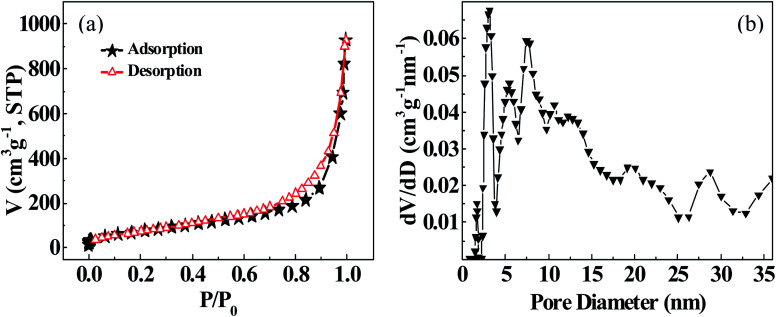
(a) TiO_2_-NTs nitrogen adsorption isotherm; (b) pore diameter distribution.

*Via* analyzing the nitrogen adsorption isotherm of TiO_2_-NTs (*cf.*Fig. 5(a)), it can be found that the adsorption isotherm could be assigned to a typical type IV adsorption curve of mesoporous materials. There exists obvious H_3_ type hysteresis loop, indicating that the TiO_2_-NTs sample contains mesoporous structures with relatively wide aperture. Moreover, the steep hysteresis loop that is almost parallel to vertical axis signifies the existence of ordered channel, which is consistent with the SEM and TEM observations. Fig. 5(b) shows the pore size distribution of TiO_2_ nanotubes (TiO_2_-NTs). The pore size distribution is mainly concentrated in the range of 3–15 nm, indicating that mesopores in TiO_2_ nanotubes occupy relatively high proportion. By fitting the experimental data, it can be determined that the specific surface area of TiO_2_-NTs is 273.2 m^2^ g^−1^; the pore volume is 929.1 cm^3^ g^−1^, and the average pore diameter is 15.6 nm.

#### 2.3.2 BET analysis of TiN-NTs

**Fig. 6:**
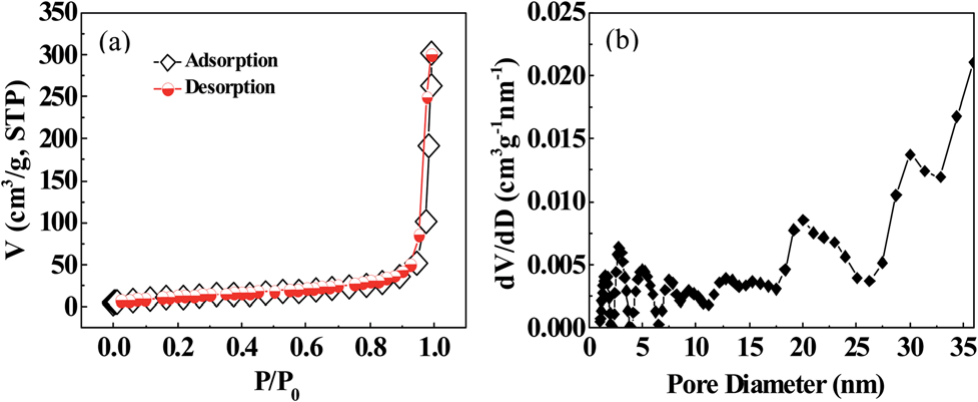
(a) TiN-NTs nitrogen adsorption isotherm; (b) pore diameter distribution.

Fig. 6 displays the BET test and analysis results for the as-fabricated TiN-NTs sample. It can be found that nitrogen adsorption isotherm of TiN-NTs still belongs to IV type curve, with the smaller H_3_ hysteresis loop. The micropores (<3 nm) have almost disappeared, and the pore size distribution has shifted from mesopore to macropore. Based on the experimental data from the Fig. 6(b), the specific surface area of TiN-NTs is calculated to be 40.9 m^2^ g^−1^, and the pore volume and average pore diameter are 301.4 cm^3^ g^−1^ and 39.5 nm, respectively. The comparison between the both sets of BET results reveals that the resulting values of TiN-NTs descend to different degrees for both specific surface area (down 5.7 times) and pore volume (down 2.1 times), but the average pore diameter increase by 1.5 times. From the context we can deduce that during the high temperature nitriding process, the micropores and some mesopores in the TiO_2_-NTs were fused and closed, but some macropores were further expanded and cracked, giving rise to the decrease of micro-mesopores and the increase of macropores in TiN-NTs.

### 2.4 Catalytic activity of Ni-NPs and Ni(*x*)@TiN-NTs

#### 2.4.1 Catalytic ability of Ni-NPs on the AB hydrolysis

**Fig. 7:**
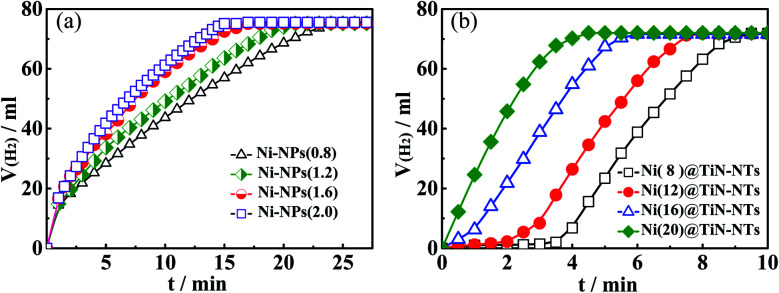
(a) Hydrolyzing AB to release H_2_ curves catalyzed by different dosages of Ni-NPs; (b) hydrolyzing AB to release H_2_ curves catalyzed by different Ni(*x*)@TiN-NTs.

According to the above catalytic activity evaluation device and specific methods, metal Ni nanoparticles (Ni-NPs) and titanium nitride nanotubes supported Ni nanoparticles (Ni@TiN-NTs) were used as catalysts, respectively, and their catalytic abilities on hydrolyzing AB to generate H_2_ at 303 K were investigated in detail. Fig. 7(a) displays the H_2_ generation curves *via* hydrolyzing AB under different Ni-NPs adding amount, where Ni-NPs(0.8), Ni-NPs(1.2), Ni-NPs(1.6) and Ni-NPs(2.0) represent the dosage of Ni-NPs in the 0.1 mol l^−1^AB solution: 0.8 g l^−1^, 1.2 g l^−1^, 1.6 g l^−1^ and 2.0 g l^−1^, respectively. Evidently, the Ni-NPs have a certain level of catalytic activity at the four dosages, and there is no catalytic induction period. With the rise of Ni-NPs addition, the H_2_ generation per unit time indicates gradual increase, making it clear that the H_2_ generation rate is proportional to the dosage of Ni-NPs. In the initial 5 min hydrolysis reaction, the H_2_ generation volume catalyzed by Ni-NPs(0.8), Ni-NPs(1.2), Ni-NPs(1.6) and Ni-NPs(2.0) are 28.5 ml, 33.7 ml, 38.4 ml and 41.2 ml, respectively, and the corresponding H_2_ mean production rates are 5.7 ml min^−1^, 6.7 ml min^−1^, 7.7 ml min^−1^ and 8.2 ml min^−1^, respectively. To reach the maximum H_2_ amount (74.5 ml), the reaction time catalyzed by Ni-NPs(2.0) is only 13 min, while it needs 24 min for Ni-NPs(0.8). Moreover, both the H_2_ release curves related to Ni-NPs(1.6) and Ni-NPs(2.0) are very close to each other, meaning that the dosage of Ni-NPs may have an upper limit. Thus, it is not difficult to infer that the optimal dosage of Ni-NPs should be controlled between 1.60 g l^−1^ and 2.00 g l^−1^.

#### 2.4.2 Catalytic ability of Ni(*x*)@TiN-NTs on the AB hydrolysis

Under the same experiment conditions as Ni-NPs, the study results about catalytic activity of Ni(*x*)@TiN-NTs are shown in Fig. 7(b). For comparison's sake, the dosage (in terms of metal Ni) of Ni(*x*)@TiN-NTs maintains the same with that of Ni-NPs(2.0). As seen in the Fig. 7(b), the four Ni(*x*)@TiN-NTs catalysts all possess better catalytic activity than Ni-NPs(2.0). Even for the weakest catalyst Ni(8)@TiN-NTs, it could also reach the top H_2_ production within 9 minutes. For Ni(8)@TiN-NTs and Ni(12)@TiN-NTs, the H_2_ release curves display evident induction period that are 3.5 min and 2 min, respectively. However, with the Ni load increasing, the induction period gradually shortens until it fully disappears. In the initial 5 min hydrolysis process, the H_2_ mean generation rates catalyzed by Ni(8)@TiN-NTs, Ni(12)@TiN-NTs, Ni(16)@TiN-NTs and Ni(20)@TiN-NTs are 5.2 ml min^−1^, 9.4 ml min^−1^, 14.5 ml min^−1^ and 14.9 ml min^−1^, respectively. Compared with Ni-NPs, the catalytic activity of the supported Ni catalyst Ni(*x*)@TiN-NTs improves significantly, which should be largely ascribed to the large specific surface area, high dispersion and excellent electron conductivity of the carrier TiN-NTs.^30^

#### 2.4.3 Effect of temperature on catalytic activity of Ni(20)@TiN-NTs

**Fig. 8:**
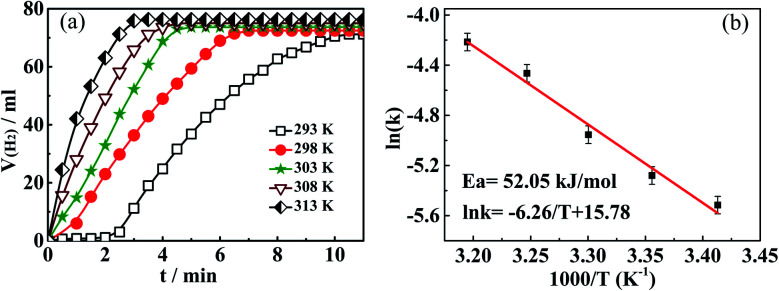
(a) Influence of temperature on catalytic performance of Ni(20)/TiN-NTs; (b) plotting of ln(*k*) against 1/*T* according to Arrhenius equation.

The reaction temperature not only affects the catalytic performance of the catalyst but also impacts the kinetic features of the hydrolysis reaction. Therefore, according to the same experimental conditions and *via* adjusting different hydrolysis temperatures (293–313 K), we further investigated the effect of temperature on the catalytic activity of Ni(20)@TiN-NTs. As shown in Fig. 8(a), it is not difficult to find that with the rise of reaction temperature, the catalytic activity of Ni(20)@TiN-NTs gradually improves, and the H_2_ generation rate markedly increases. This is, temperature is positively correlated with the rate of hydrolysis reaction. At the highest temperature (313 K), the complete H_2_ release can be achieved in less than 3 min. However, the existence of induction period at the lowest temperature (293 K) slightly reduce its catalytic ability; after passing the induction period, the H_2_ release rate can still grows rapidly. The above findings and analysis manifest that temperature holds different degrees of impact on the catalytic performance of Ni(20)@TiN-NTs, which is expected to be used for purposively regulating the hydrogen release rate.

In order to calculate the turnover frequency (TOF) for the catalyst Ni(20)@TiN-NTs, we here suggest a concise but reasonable equation:^26^ TOF = [*P* × *V*_H2_/*RT*]/*n*_M_ × *t*, where *P* denotes the local atmospheric pressure; *V*_H2_ is the H_2_ volume generated in given reaction time; *R* and *T* stand for the universal gas constant and reaction temperature, respectively; *n*_M_ and *t* separately represent the mole number of the metal in the catalyst and the given reaction time. According to the above temperature-dependent experimental results, the turnover frequency (TOF) at the room temperature (298 K) in the initial 3 min reaction time can be determined to be 11.73 mol(H_2_) (mol Ni)^−1^ min^−1^, or 294268 mol(H_2_) (mol Ni)^−1^ min^−1^.

Allowing for the far outweighing amount of H_2_O than that of AB, the concentration of H_2_O can be regarded to be unchanged during hydrolysis, and the hydrolysis reaction of AB could thus be viewed as a one-reactant type. On base of the principle of chemical kinetics, we plot a graph of ln(*V*_H2_) against the initial reaction time *t*, and get a good linear relationship. This result surely reveals that the AB hydrolysis reaction accords with the first order dynamic characteristics, which is consistent with our previous studies.^31^ From the straight slopes, we can get a series of reaction rate constants (*k*) that corresponds to the different temperatures. In accordance to Arrhenius equation, plotting natural logarithm ln(*k*) to the reciprocal (1/*T*) of temperature *T* provides an approximate line with a slope of −6.26 (*cf.*Fig. 8(b)), by means of which the apparent activation energy *E*_a_ can be determined to be 52.05 kJ mol^−1^. In line with the comparison of the activation energy, the key indicator for catalytic kinetics, it is definitely judged that the catalytic ability of Ni(20)@TiN-NTs surpasses some transition metals or their alloys (*e.g.*, NiCu@C,^32^ Cu_0.1_@Co_0.45_Ni_0.45_/graphene^33^), even some noble metal-based catalyst (*e.g.*, Pt/C (2wt%),^34^ PtNi@SiO_2_,^35^ PtPd NPs,^36^ PtNiO/NGO,^37^ Pt@SiO_2_ (ref. 38)).

#### 2.4.4 Durability test of Ni(20)@TiN-NTs

**Fig. 9:**
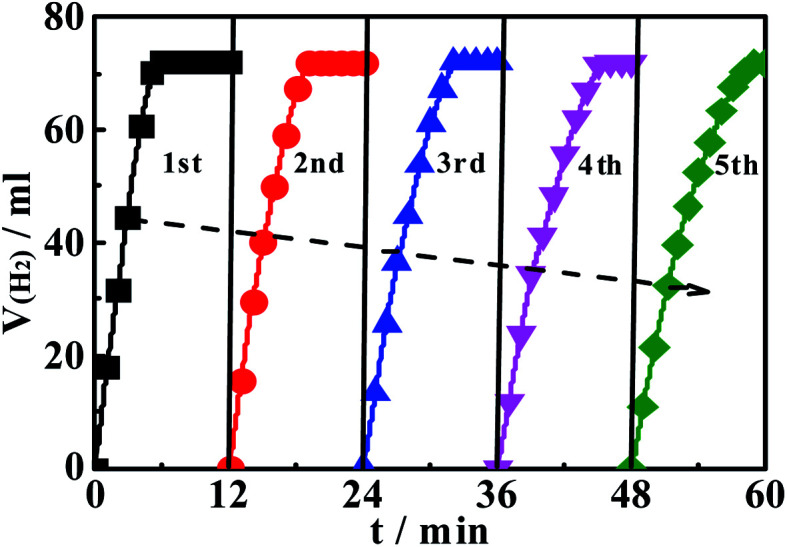
The durable test of Ni(20)/TiN-NTs for 5 runs of repeated use.

The service life or durability is one of the three main performances (activity, selectivity and stability) of the catalyst, and is also a necessary parameter in the practical application process. Here, by using Ni(20)@TiN-NTs as the catalyst, we conduct 5 cycling experiments under the same conditions as above, and the results are shown in Fig. 9. It can be seen that under the temperature of 303 K and with the increase of times of recycling, the activity of the catalyst decreases gradually. After the fifth cycle, it could still remain 87% of the initial catalytic activity, which signifies that Ni(20)@TiN-NTs has excellent application stability or reliable durability. The possible reasons for the reduction of catalytic activity after repeated use could be ascribed as: (1) during the catalytic reaction process, the by-product of the hydrolysis of ammonia borane gradually precipitates and adsorbs on the surface of the catalyst, covering part of the active sites of the catalyst; (2) during the collection, washing and reuse of the catalyst, Ni particles fall off the surface of the carrier TiN-NTs, causing a small amount of the active ingredients to be lost.

At last, it is very essential for us to point out that no any NH_3_ or similar species were detected during all the above hydrolytic process, reflecting the satisfactory catalytic selectivity for the Ni(20)@TiN-NTs catalysts from one aspect.

## 3 Conclusion

We here put forward a flexible and efficient hydrothermal-nitriding tactics for fabricating titanium nitride nanotubes (TiN-NTs), and deposit nanosized Ni particles on surface of the TiN-NTs *via* PVP-mediated non-aqueous phase reduction–deposition route, to obtain the supported catalyst Ni@TiN-NTs. The comprehensive characterization results manifest that the as-fabricated TiN-NTs takes hollow short tubular shape with apparent mean diameter of 130 nm, and the length of the tube is obviously shorter than the precursor TiO_2_-NTs, with the tube surface full of pores. The tiny Ni particles attached to the surface of TiN-NTs freely link with each other, resulting in the unordered, porous and diverse morphology. The specific surface area and average pore diameter of TiN-NTs are, respectively, 40.9 m^2^ g^−1^ and 39.5 nm, and especially the later presents an increase by 1.5 times compared with the precursor TiO_2_-NTs. It is the unique microstructure of Ni(*x*)@TiN-NTs that can provide larger specific surface area, more active sites and channels, availing the enhancement of catalytic activity. The supported Ni catalysts Ni(*x*)@TiN-NTs hold better catalytic activity than the metal Ni nanoparticles (NPs(2.0)), which should be largely ascribed to the larger specific surface area, high dispersion and excellent electron conductivity of the carrier TiN-NTs. With the rise of hydrolysis reaction temperature, the catalytic activity of Ni(*x*)@TiN-NTs gradually improves, and the H_2_ release rate markedly increases, signifying the positive correlation effect of temperature with the hydrogen evolution reaction. The existence of induction period at the lower temperature (293 K) and low Ni loading amount (Ni(8)@TiN-NTs, Ni(12)@TiN-NTs) could be expected to be used for purposively regulating the hydrogen release rate. The Ni supported catalyst Ni(20)@TiN-NTs has the highest catalytic activity among the involved catalysts, with the total turnover frequency of 11.73 mol(H_2_) (mol Ni)^−1^ min^−1^, and apparent activation energy of 52.05 kJ mol^−1^ at 303 K. After 5 using cycles, the Ni(20)@TiN-NTs catalyst still maintained 87% of the initial catalytic activity. Theoretically, the preparing strategy for TiN-NTs and Ni(*x*)@TiN-NTs can also be extended to the fabrication of other metal or alloy supported catalysts, with great potential development prospects.

## Data availability

The specific experimental data used for depicting the figures of this study are available from the corresponding author upon request.

## Conflicts of interest

The authors declare no competing financial interest.

## Supplementary Material

RA-010-D0RA06920E-s001

## References

[cit1] Veras S., Mozer S., Danielle S. (2016). Hydrogen: trends, production and characterization of the main process worldwide. Int. J. Hydrogen Energy.

[cit2] Andrews J., Shabani B. (2014). The role of hydrogen in a global sustainable energy strategy. Wiley Interdiscip. Rev.: Energy Environ..

[cit3] Mudita N., Rita K. (2018). An evolving energy solution: intermediate hydrogen storage. Int. J. Hydrogen Energy.

[cit4] Zhang J., Duan Y., Zhu Y., Wang Y., Yao H., Mi G. (2017). Evenly dispersed microspherical amorphous alloy Co_*x*_B_1−*x*_: robust and magnetically recyclable catalyst for alcoholyzing ammonia borane to release H_2_. Mater. Chem. Phys..

[cit5] Yao K. S., Zhao C. C., Wang N., Li T. J., Lu W. W., Wang J. J. (2020). An aqueous synthesis of porous PtPd nanoparticles with reversed bimetallic structures for highly efficient hydrogen generation from ammonia borane hydrolysis. Nanoscale.

[cit6] Fu F., Wang C., Wang Qi, Martinez-Villacorta A. M. (2018). Highly selective and sharp volcano-type synergistic Ni_2_Pt@ZIF-8-Catalyzed hydrogen evolution from ammonia borane hydrolysis. J. Am. Chem. Soc..

[cit7] Li H. Z., Wang P. Y., Chen X. N. (2014). Ammonia borane: a high capacity chemical hydrogen storage material. Chin. Sci. Bull..

[cit8] Zhang J., Li H., Zhang H., Zhu Y., Mi G. (2016). Porously hierarchical Cu@Ni cubic-cage microstructure: very active and durable catalyst for hydrolytically liberating H_2_ gas from ammonia borane. Renewable Energy.

[cit9] Zhang J., Dong Y., Wang Y., Zhu Y., Duan Y. (2018). A novel route to synthesize hydrogen storage material ammonia borane *via* copper(ii)–ammonia complex liquid phase oxidization. Int. J. Energy Res..

[cit10] Sen B., Esra K., Buse D., Tugba O. O. (2018). Highly efficient polymer supported monodisperse ruthenium–nickel nanocomposites for dehydrocoupling of dimethylamine borane. J. Colloid Interface Sci..

[cit11] Matteo M., Tiziano M., Emiliano F., Matteo C. (2018). Nanostructured Pd/Pt nanoparticles: evidences of structure/performance relations in catalytic H_2_ production reactions. Appl. Catal., B.

[cit12] Peng S., Liu J., Zhang J., Wang F. (2015). An improved preparation of graphene supported ultrafine ruthenium(0) NPs: very active and durable catalysts for H_2_ generation from methanolysis of ammonia borane. Int. J. Energy Res..

[cit13] Xu P., Lu W. W., Zhang J., Zhang L. (2020). Efficient Hydrolysis of Ammonia Borane for Hydrogen Evolution Catalyzed by Plasmonic Ag@Pd Core–Shell Nanocubes. ACS Sustainable Chem. Eng..

[cit14] Park M. S., Jin Kyu K., Han T.-S. (2020). Impregnation approach for poly(vinylidene fluoride)/TiN oxide nanotube composites with high tribological performance. J. Mater. Sci. Technol..

[cit15] Brock D., Yi Li, Andrei M. (2019). Plasmon-Enhanced Electron Harvesting in Robust Titanium Nitride Nanostructures. J. Phys. Chem. C.

[cit16] Peng X., Huo K. F., Fu J. J. (2013). Coaxial PANI/TiN/PANI nanotube arrays for high-performance supercapacitor electrodes. Chem. Commun..

[cit17] Negar M., Venkateswara Rao C., Salley S. O. (2016). Nanostructured titanium nitride as a novel cathode for high performance lithium/dissolved polysulfide batteries. J. Power Sources.

[cit18] Xiao Y., Zhan G., Fu Z. (2014). Robust non-carbon titanium nitride nanotubes supported Pt catalyst with enhanced catalytic activity and durability for methanol oxidation reaction. Electrochim. Acta.

[cit19] Lu W., Zhang Y., Zhang J., Xu P. (2020). Reduction of Gas CO_2_ to CO with High Selectivity by Ag Nanocube-Based Membrane Cathodes in a Photoelectrochemical System. Ind. Eng. Chem. Res..

[cit20] Mosavati N., Chitturi V. R., Salley S. O. (2016). Nanostructured titanium nitride as a novel cathode for high performance lithium/dissolved polysulfide batteries. J. Power Sources.

[cit21] Yao K., Zhao C., Sun N., Lu W., Zhang Y., Wang H., Wang J. (2017). Freestanding CuS nanowalls: ionic liquid-assisted synthesis and prominent catalytic performance for the decomposition of ammonium perchlorate. CrystEngComm.

[cit22] Wen Z., Cui S., Pu H. (2011). Metal nitride graphene nanohybrids general synthesis and multifunctional titanium nitride grapheme electrocatalyst. Adv. Mater..

[cit23] Grote F., Zhao H., Lei Y. (2015). Self-supported carbon coated TiN nanotube arrays innovative carbon coating leads to an improved cycling ability for supercapacitor applications. J. Mater. Chem. A.

[cit24] Pan Z., Xiao Y., Fu Z., Zhan G., Wu S. (2014). Hollow and porous titanium nitride nanotubes as high-performance catalyst supports for oxygen reduction reaction. J. Mater. Chem. A.

[cit25] Ramachandran P. V., Gagare P. D. (2007). Preparation of ammonia borane in high yield and purity, methanolysis, and regeneration. Inorg. Chem..

[cit26] Zhang J., Dong Y., Liu Q., Zhou M., Mi G., Du X. (2019). Hierarchically alloyed Pd-Cu microarchitecture with tunable shapes: morphological engineering, and catalysis for hydrogen evolution reaction of ammonia borane. Int. J. Hydrogen Energy.

[cit27] Yang C., Wang H., Lu S., Wu C. (2015). Titanium nitride as an electrocatalyst for V(ii)/V(iii) redox couples in all-vanadium redox flow. Electrochim. Acta.

[cit28] Thotiyl M., Ottakam M., Kumar T. R., Sampath S. (2010). Pd Supported on Titanium Nitride for Efficient Ethanol Oxidation. J. Phys. Chem. C.

[cit29] Musthafa Muhammed O. T., Srinivasan S. (2008). High performance platinized titanium nitride catalyst for methanol oxidation. Chem. Commun..

[cit30] Byun J., Ahn S. H., Kim J. J. (2020). Self-terminated electrodeposition of platinum on titanium nitride for methanol oxidation reaction in acidic electrolyte. Int. J. Hydrogen Energy.

[cit31] Zhang J., Wang Y., Zhu Y., Mi G., Du X. G., Dong Y. N. (2018). Shape-selective fabrication of Cu nanostructures: contrastive study of catalytic ability for hydrolytically releasing H2 from ammonia borane. Renewable Energy.

[cit32] Yousef A., Barakat N. A. M., El-Newehy M., Kim H. Y. (2012). Stable electrospun NiCu nanorods@carbon nanofibers for highly efficient dehydrogenation of ammonia borane. Int. J. Hydrogen Energy.

[cit33] Meng X., Yang L., Cao N., Du C., Hu K., Su J., Luo W., Cheng G. (2014). Graphene-supported trimetallic core–shell Cu@CoNi nanoparticles for catalytic hydrolysis of amine borane. ChemPlusChem.

[cit34] Xu Q., Chandra M. (2007). A portable hydrogen generation system: catalytic hydrolysis of ammonia-borane. J. Alloys Compd..

[cit35] Qi X., Li X., Chen Bo, Lu H., Wang Le (2016). Highly Active Nanoreactors: Patchlike or Thick Ni Coating on Pt Nanoparticles Based on Confined Catalysis. ACS Appl. Mater. Interfaces.

[cit36] Amali A. J., Aranishi K., Uchida T., Xu Q. (2013). PdPt Nanocubes: A High-Performance Catalyst for Hydrolytic Dehydrogenation of Ammonia Borane. Part. Part. Syst. Charact..

[cit37] Zhao B., Kun F., Wang Y., Lv X., Zheng H. (2017). Pt_*x*_Ni_10−*x*_O nanoparticles supported on N-doped graphene oxide with a synergetic effect for highly efficient hydrolysis of ammonia borane. Catal. Sci. Technol..

[cit38] Hu Y., Wang Y., Lu Z.-H., Chen X., Xiong L. (2015). Core–shell nanospheres Pt@SiO_2_ for catalytic hydrogen production. Appl. Surf. Sci..

