# Supplementary figures and images for: TiN nanotube supported Ni catalyst Ni@TiN-NTs: experimental evidence of structure–activity relations in catalytically hydrolyzing ammonia borane for hydrogen evolution
Source: RSC Adv. 2020 Oct 8;10(61):37209–17. doi: 10.1039/d0ra06920e (PMC9057120; doi:10.1039/d0ra06920e)

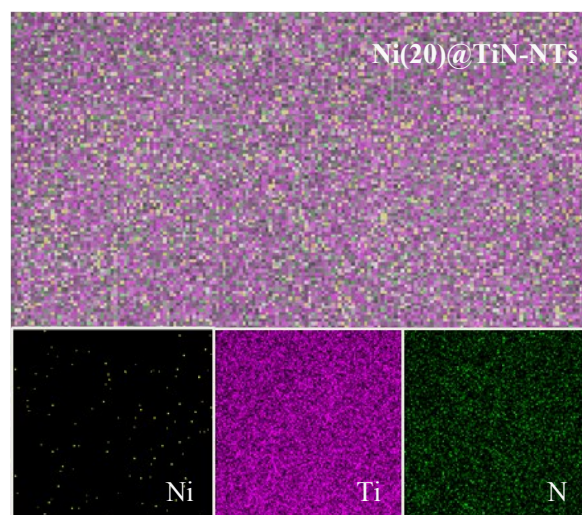

Fig.S1 SEM - mapping image of as-fabricated catalyst Ni(20)@TiN-NTs

Supplement: RA-010-D0RA06920E-s001 [file RA-010-D0RA06920E-s001.pdf]
